# Synbiotic therapy decreases microbial translocation and inflammation and improves immunological status in HIV-infected patients: a double-blind randomized controlled pilot trial

**DOI:** 10.1186/1475-2891-11-90

**Published:** 2012-10-29

**Authors:** Luz A González-Hernández, Luis F Jave-Suarez, Mary Fafutis-Morris, Karina E Montes-Salcedo, Luis G Valle-Gutierrez, Ariel E Campos-Loza, Luis Fermin Enciso-Gómez, Jaime F Andrade-Villanueva

**Affiliations:** 1 HIV Unit Hospital Civil de Guadalajara “Fray Antonio Alcalde”, University of Guadalajara, Calle Hospital 278, Colonia Alcalde Barranquitas, Guadalajara, Jalisco, 44280, Mexico; 2 Centro Universitario de Ciencias de la Salud (CUCS), UdeG, Guadalajara, Jalisco, Mexico; 3 Centro de Investigación Biomédicas de Occidente (CIBO), Instituto Mexicano del Seguro Social (IMSS), Guadalajara, Jalisco, Mexico

## Abstract

**Background:**

HIV-infection results in damage and dysfunction of the gastrointestinal system. HIV enteropathy includes pronounced CD4+ T-cell loss, increased intestinal permeability, and microbial translocation that promotes systemic immune activation, which is implicated in disease progression. A synbiotic is the combination of probiotics and prebiotics that could improve gut barrier function. Our study goal was to determine whether the use of a synbiotic, probiotics or a prebiotic can recover immunological parameters in HIV-infected subjects through of a reduction of microbial translocation and pro-inflammatory cytokine production.

**Methods:**

A randomized, double-blind controlled study was performed; twenty Antiretroviral treatment-naïve HIV-infected subjects were subgrouped and assigned to receive a synbiotic, probiotics, a prebiotic, or a placebo throughout 16 weeks.

**Results:**

We had no reports of serious adverse-events. From baseline to week 16, the synbiotic group showed a reduction in bacterial DNA concentrations in plasma (*p* = 0.048). Moreover, the probiotic and synbiotic groups demonstrated a decrease in total bacterial load in feces (*p* = 0.05). The probiotic group exhibited a significant increment of beneficial bacteria load (such as *Bifidobacterium*; *p* = 0.05) and a decrease in harmful bacteria load (such as *Clostridium; p* = 0.063). In the synbiotic group, the CD4+ T-cells count increased (median: +102 cells/μL; *p* = 0.05) and the level of Interleukin 6 cytokine decreased significantly (*p* = 0.016).

**Conclusions:**

Our study showed a significant increase in CD4+ T lymphocyte levels in the synbiotic group, which could delay the initiation of antiretroviral therapy and decrease costs in countries with limited resources.

## Introduction

A huge Gastrointestinal (GI) pathology is observed in patients infected with HIV even during primary infection. Approximately 60% of total CD4+ T cells, reside in Gut-associated lymphoid tissue (GALT), and of all tissues, the latter is one of the most strongly affected during HIV infection [1]. In 1984, Kotler and collaborators described HIV enteropathy; subsequently, several studies have demonstrated HIV-associated damage to the GI tract [2-4].

### Gastrointestinal damage in HIV infection and microbial translocation

Once HIV enters the mucosa of the gut, it finds a large pool of resting Ki67-CD4+ T cells; up to 60% of these cells are infected and are capable of produce the virus, constituting a dense network of cells in the intestinal mucosa, which is capable of spreading the infection to uninfected cells through cell-to-cell contact. This spread allows the maintenance of a continuous chain of viral transmission and forms part of a large reservoir that is currently impossible to eradicate. On the other hand, activated CD4+ T cells (effector) produce high viral-load levels, generating high viral gradient in the mucous membrane tissues, resulting in infection amplification and spread over long distances [5].

HIV exerts a direct effect on enterocytes. The viral tat protein inhibits glucose uptake by the enterocytes, altering their function; the gp120 protein increases enterocyte intracellular calcium and causes tubulin depolymerization, in turn causing cytoskeleton dysfunction, with destabilization of intercellular junctions and further increased intestinal permeability. There is a simultaneous occurrence of a decrease in the expression of genes involved in epithelial mucosal repair (such as trefoils) and increased immune activation genes, leading to high levels of pro-inflammatory cytokines as Tumor necrosis factor (TNF)-α, Interferon (INF)-γ, and Interleukin (IL)-6, -8, and −12 [2,6].

The enteropathy associated with HIV-infection is characterized by villous atrophy, crypt hyperplasia, vitamin B12 and bile acid malabsorption, epithelial cell damage, with enterocyte apoptosis and disruption of tight and adherent junctions with an increase in intestinal permeability; moreover, depletion of Peyer’ s patches as well as of CD4+ T cells in lamina propria combined with a reduced ability to produce secretory IgA and defensins comprise conditions that favor persistent microbial overgrowth in the intestinal lumen, microbial translocation with increased plasma levels of Lipopolysaccharides (LPS), and 16S rRNA, which can provoke the development of chronic local and systemic immune stimulation that may lead to disease progression [2,7-11].

### Effect of microbiota on the immune system

Microbiota of intestinal tract constitute a complex ecosystem of microorganisms; there are from 10–100 trillion microorganisms that populate the adult intestine and these provide some benefits to the host [12]. HIV infection alters gut microbial ecology [13]. Several probiotic organisms and prebiotic agents have shown to enhance intestinal epithelial barrier functions, reduce inflammation, and support effective Th-1 responses [14].

Synbiotics are the combination of probiotics plus prebiotics. The definition of probiotics is live microbial feed supplements that confer health benefits when administered in adequate amounts (10^7^-10^11^ colony forming units (cfu)) and are classified by the US Food and Drug Administration (FDA) as “live biotherapeutics” when utilized in humans [15,16]. Probiotics exert an effect on the immunological response; they mainly stimulates polymeric IgA secretion, avoid bacterial overgrowth and their translocation, and produce a self-limited inflammatory response through development of regulatory T (Treg) cells by anti-inflammatory cytokine production [12,15,17-19].

Prebiotics are non-digestible oligosaccharides that selectively stimulate the growth of some bacteria, altering the composition and metabolic activity of gut microbiota [20]. A study in mice demonstrated that administration of prebiotics such as FructoOligoSaccharides (FOS) exerts an immunostimulating effect on inductive sites such as Peyer’s patches [21]. Inulin-type fructans are non-digestible oligosaccharides with prebiotic properties and can be obtained from agave plants [22,23].

The diversity of GI microbiota is directly related with the number of nutrients [24] and lower beneficial bacteria counts in feces have been reported in HIV-infected subjects compared with healthy controls [13].

Patients with HIV suffer immunological and structural defects in GI tract; thus, new interventions in HIV-positive patients are necessary to restore the integrity of the epithelial and immune system of GALT and block the ways through which microbial products cause chronic immune activation. Therefore, we evaluated the use of a synbiotic, probiotics and a prebiotic to expand beneficial microbiota that aid in decreasing bacterial translocation and pro-inflammatory cytokine production, thereby improving immune functions in HIV-infected subjects.

## Materials and methods

### Study design and participants

A randomized, prospective, double-blind controlled pilot study was conducted in HIV-infected adult patients who were divided into four groups to receive the following: group I: probiotics; group II: the synbiotic; group III: the prebiotic, and group IV: the placebo. The patients were recruited from August 2008 through June 2009 at the HIV Unit Department of Hospital Civil de Guadalajara Fray Antonio Alcalde.

Patients were randomly assigned to receive probiotics, a synbiotic, a prebiotic, or placebo during 16 weeks; all products (gel formulation) were made by biotechnology company called Biotek in Guadalajara city, they required refrigeration and were taken once daily. We used 10 g of agavins from *Agave tequilana* Weber var. azul as the prebiotic; these types of fructans comprise a complex mixture of FOS containing mainly β(2–1) linkages, and some β(2–6) linkages, with branches and a glucose moiety [23]. As probiotics, we employed *Lactobacillus rhamnosus* HN001 plus *Bifidobacterium lactis* Bi-07 at 10^9^ cfu/mL, and the synbiotic was the combination of probiotics plus prebiotic, the placebo was a product of Biogel without the two types of probiotics and without the prebiotic, but with the same flavor and characteristics. The Institutional Review Board at the study site approved the study, and all patients were enrolled immediately after providing written informed consent.

Inclusion criteria included the following: documented HIV infection; age between 18 and 65 years; negative for Hepatitis B and C viruses; Antiretroviral (ARV) treatment-naïve, in stage A or B according to the U.S. Centers for Disease Control and Prevention (CDC) classification system, with a CD4+ count of >350 cells/mm^3^. Patients were not included in the study if they were pregnant, had hepatic or renal insufficiency, cancer, immunotherapy, use of antibacterial therapy (for >15 days), had knowledge of an allergy to bovine milk proteins or if the dose compliance with the gel formulation was <95%.

### Safety parameters and assessment of quality of life (QoL) assessment

Participating patients were requested to check their body temperature, to report immediately to the researcher for possible symptoms of infections, and were examined by means of laboratory and physical testing at least once monthly.

The Irritable Bowel Syndrome-Quality of life (IBS-QoL) questionnaire in a local language was utilized to evaluate the impact of gastrointestinal symptoms or treatment complications at baseline and at 16-week visits. The IBS-QoL is a self-administered instrument employed to derive an overall score with a 34-item, condition-specific questionnaire that has been approved for identifying GI-related QoL measures in HIV-infected patients [25,26], in whom highest scores signify poorest IBS-related QoL in our study.

### Immunological variables determined at baseline and at week-16 visits

#### Absolute CD4+ T-lymphocyte count

Absolute CD4+ T-cell counts were quantified as absolute concentration (cells per cubic mm) by means of flow cytometry at a state-certified laboratory.

#### Cytokines levels

We measured IL-10 concentrations in plasma as anti-inflammatory cytokine, and pro-inflammatory cytokines IL-1β, IL-6, and TNF-α by ELISA using commercially available kits (R&D systems). Results were expressed in units of pg/mL.

### Molecular analysis

#### Viremia quantification

HIV-1 RNA was measured in plasma at baseline and at week 16, with the AMPLICOR HIV-1 MONITOR ultrasensitive assay (Roche Diagnostics, version 1.0) with a lower detection limit of 50 copies/mL at a state-certified laboratory.

#### Purification of bacterial DNA in plasma and stool

From 200 μL of plasma and from 200 g of frozen stool samples, bacterial DNA was extracted employing the DNeasy Blood and Tissue Kit and QIAamp DNA Stool Mini Kit (Qiagen), respectively, both according to the manufacturer’s instructions. DNA concentrations were determined with a DU 640 spectrophotometer.

#### Quantitative (q) PCR for bacterial 16S rRNA in plasma and feces

Bacterial translocation was measured by means of 16S rRNA levels in plasma, and 16S rRNA levels in feces were utilized to determinate total bacterial load in stool. We used a final volume of 10-μL amplification reaction consisting of 2 μL of 1X PCR buffer (Light Cycler Fast Start DNA Master Plus Reaction Mix SYBRGreen I, Roche), 5 pmol forward and 5 pmol reverse primers and 2 ng of template plasma DNA or template feces DNA (for total bacterial load in feces). We employed the following primer pairs: forward (8 F: 5′-AGT TTG ATC CTG GCT CAG-3) and reverse (515R: 5′-GWA TTA CCG CGG CKG CTG-3′) primers to amplify bacterial DNA templates encoding 16S rRNA [10,27]. We amplified the DNA in triplicate and calculated mean values. The count was absolute using a standard curve with the four dilutions of concentrations known of a standard plasmid DNA. Conditions for the DNA amplification reaction comprised 95°C for 10 min, followed by 40 cycles at 95°C for 10 s, 60°C for 10 s, and 72°C for 22 s. Thereafter a melting temperature analysis was performed as follows: at 95°C for 10 s; at 60°C for 1 min, and by slowly heating the sample to 95°C at a temperature rate of 0.1°C/s.

#### Quantitative PCR (qPCR) for Bifidobacterium and Clostridium spp. in feces

To detect changes in stool bacterial composition at baseline and at week 16, we measured the bacterial loads, dividing as beneficial bacteria the *Bifidobacterium* spp. Load, and as harmful bacteria, the *Clostridium* spp. load. The primer pairs used to detect *Bifidobacterium* spp. were the following: forward 5′-CGC GTC YGG TGT GAA AG-3′and reverse 5′-CCC CAC ATC CAG CAT CCA-3′ [27,28]. The primer pairs to quantify *Clostridium* spp. were forward 5′-AAA TGA CGG TAC CTG ACT AA-3′ and reverse 5′-CTT TGA GTT TCA TTC TTG CGA A-3′ [27,29]. All qPCR reactions were performed in a final volume of 10 μL consisting of 2 μL of 1X PCR buffer (Light Cycler Fast Start DNA Master Plus Reaction Mix SYBRGreen I, Roche), 5 pmol of forward and 5 pmol of reverse primers, and 2 ng of template feces DNA. The following amplification conditions were employed: 95°C for 10 min; followed by 40 cycles at 95°C for 15 s, at 65°C or 60°C (for *Clostridium* or *Bifidobacterium*, respectively) for 30 s, and at 72°C for 10 s. Additionally, melting temperature analysis was performed as follows: at 95°C for 10 s; at 60°C for 1 min, and by slowly heating the sample to 95°C at a temperature rate of 0.1°C/s.

### Statistical analysis

Comparisons among all groups were carried out by performing the Kruskal-Wallis and the Mann–Whitney *U* tests. Correlations were analyzed utilizing Spearman rank-correlation for nonparametric data. Other statistical tests employed are indicated in the text. All statistical analysis was undertaken using SPSS version 14 software.

## Results

### Subject characteristics

**Figure 1:**
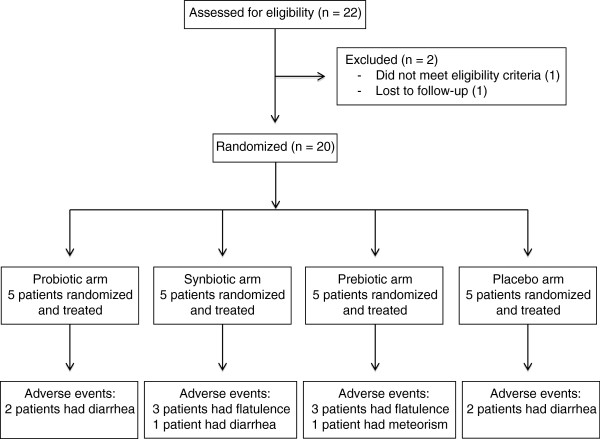
**Study enrollment.** Twenty-two patients were screening, two of them were excluded before the randomized visit, and the remainder completed the 16 weeks of treatment.

The study included 20 patients, with five participants for each of four groups. Characteristics of the patients in the four groups were similar and are depicted in Table 1. They did not show malnutrition or wasting syndrome, and metabolic variables fell within normal ranges at all times. IBS-QoL scores were similar and did not worsen during the study in either treatment arm (baseline data are shown in Table 1). Study enrollment is shown in Figure 1, during the study, some patients showed an increment of flatulence, diarrhea and meteorism, without statistical significance.

**Table 1:** Demographic and clinical baseline characteristics of as-treated patients

**Characteristics**	**Probiotic n = 5**	**Synbiotic n = 5**	**Prebiotic n = 5**	**Placebo n = 5**
Age, Mean ± SD	28 ± 6	26 ± 7	27 ± 6	30 ± 8
Gender				
Male	4	5	4	5
Female	1	0	1	0
Race (%)				
Hispanic	5 (100)	5 (100)	5 (100)	5 (100)
Body mass index (kg/m2) Mean ± SD	24 ± 2	23 ± 6	25 ± 6	24 ± 6
Clinical stage (%)	Stage A: 15	Stage A: 14	Stage A: 10	Stage A: 18
	Stage B: 85	Stage B: 86	Stage B: 90	Stage B: 82
HIV RNA level (copies/mL), median	68100	56881	40800	54460
CD4 cell count (cells/mm^3^), mean	754	620	533	542
Cholesterol (mg/dL) Mean ± SD	137 ± 16	161 ± 32	179 ± 53	148 ± 13
Triglycerides (mg/dL) Mean ± SD	88 ± 27	105 ± 27	149 ± 65	118 ± 79
IBS-QoL scale score	34	36	35	34

### Treatment compliance

In all groups, we observed an adherence upper than 98%, with good tolerance and we did not identify serious side effects that forced us to suspend the regimen.

### Bacterial translocation and bacterial stool composition

**Figure 2:**
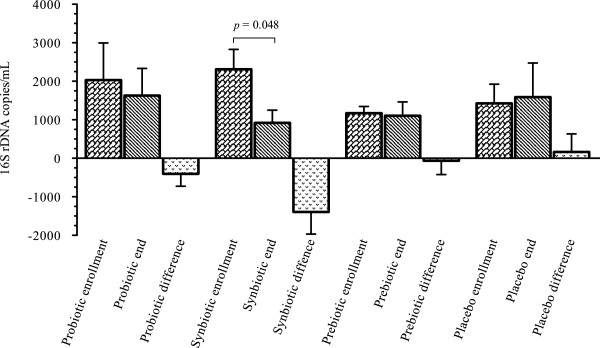
**Microbial translocation.** Quantification of bacterial 16 s rDNA extracted from plasma in the different groups. The graph shows the differences found in the four study groups at enrollment and the end of the study, in which the only group to reach statistical significance was the synbiotic group. Data shown in mean ± Standard error of the mean (SEM).

**Figure 3:**
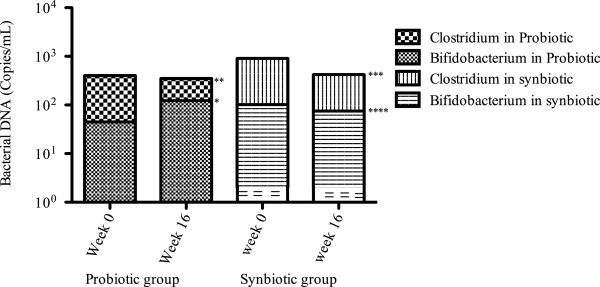
**Bacterial composition in feces.** Quantification of bacterial composition in feces in probiotic and synbiotic groups. The graph shows the differences from baseline to week 16. The probiotic group demonstrated an increase in beneficial bacterial DNA levels achieving statistical significance (*p = 0.049) and a tendency of decrease in harmful bacterial DNA levels (**p = 0.063). The synbiotic group had a tendency of decrease both type of bacterias ***p = 0.082, ****p = 0.091. Data shown in mean.

A reduction in bacterial DNA concentrations in plasma was observed in the probiotics, synbiotic, and prebiotic groups, but statistical significance was reached only in the synbiotic group (*p* = 0.048) (Figure 2). Additionally, the probiotic and synbiotic groups demonstrated a decrease in total bacterial load in feces (*p* = 0.045 and 0.048, respectively). The prebiotic group showed a tendency toward higher bacterial load in feces (data non shown). On the other hand, some differences in bacterial stool composition were found between groups; the probiotic group exhibited a significant increase of microbial load of *Bifidobacterium* spp. (*p* = 0.049) and a decrease in *Clostridium* spp. (*p* = 0.063), while the synbiotic group demonstrated a reduction non-significant in beneficial and harmful bacterial loads (*p* = 0.091 and *p* = 0.082, respectively) (Figure 3). The prebiotic and placebo groups had similar behaviour with an increased level of *Bifidobacterium* and *Clostridium* load (not statistically significant) (data not shown).

### Immunological parameters

#### Lymphocyte CD4+ T-cell count

**Figure 4:**
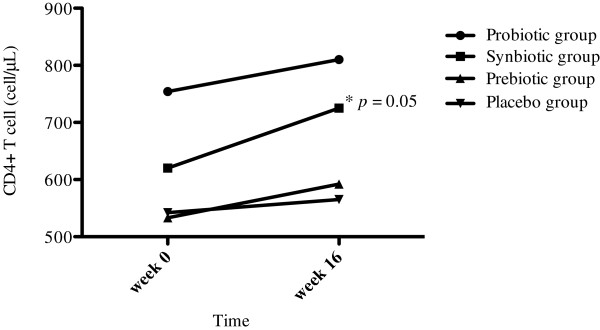
**Quantification of CD4+ T cells.** Quantification of CD4+ T cells in peripheral blood in all groups, before and after treatment. The graph shows that the synbiotic group had a statistically significant elevation in CD4+ T cell levels (median: +102 cells/μL).

At the end of the study, all patients under treatment showed an increase of CD4+ T lymphocytes; however, the synbiotic group had greater increases in CD4+ T-cell count (mean, +102 cells; *p* = 0.05) (Figure 4). No patient merited exclusion due to a low level CD4+ T-cell count and to the need to initiate ARV therapy; no patient had a diagnosis of opportunistic infections.

#### Measurement of cytokines

**Figure 5:**
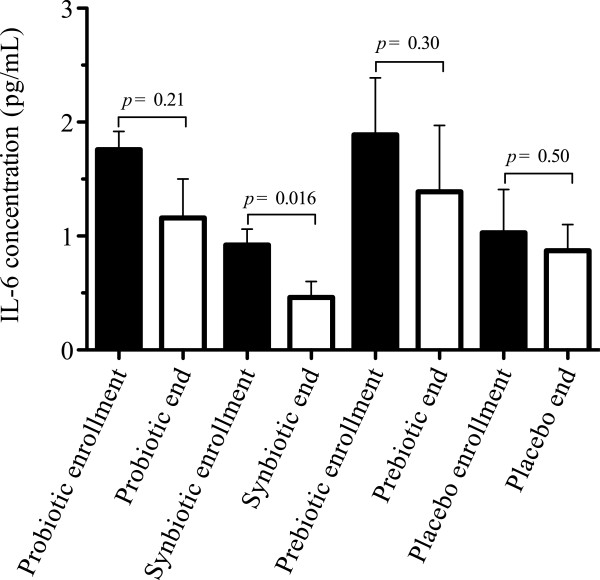
**Levels of Interleukin 6 (IL-6) in all groups.** Concentration of IL-6 in plasma at baseline and at week 16. The decrease of IL-6 in synbiotic group was statistically significant (*p* = 0.016), changes in the other groups were not significant. Data shown in mean ± Standard error of the mean (SEM).

**Figure 6:**
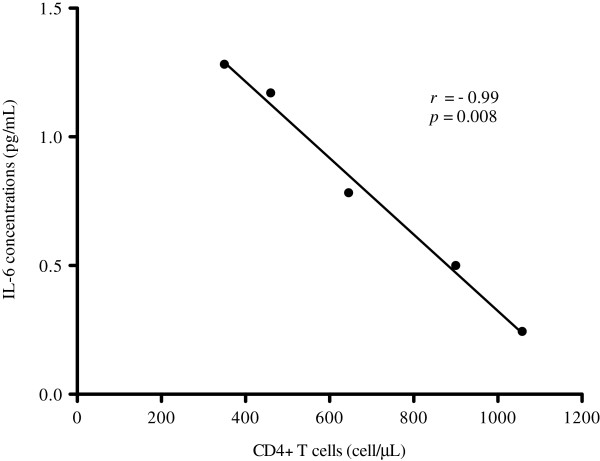
**Correlation between Interleukin 6 (IL-6) levels in plasma and CD4+ T-cell count in peripheral blood.** Using the Spearman test, a negative correlation between IL-6 concentration in plasma and absolute CD4+ T-cell number in peripheral blood was demonstrated in the placebo group, with statistical significance (*r* = −0.99; *p* = 0.008).

Similar levels of TNF-α, IL-1β, and IL-10 cytokines were found in all groups during enrollment and at the end of the study (data not shown). The IL-6 cytokine level decreased significantly in the synbiotic group (*p* = 0.016) (Figure 5) and a negative correlation between IL-6 levels and CD4+ T-cells was revealed in the placebo group (*r* = −0.99; *p* = 0.008) (Figure 6).

## Discussion

Within the immunopathogenesis of HIV infection, there is a state of constant systemic immune activation that is attributed in part to the enteropathy caused by HIV itself and to the translocation of microbes and/or microbial products from the intestinal lumen into the circulation [8,30].

We evaluated the efficacy and safety of *Lactobacillus rhamnosus* HN001 plus *Bifidobacterium lactis* Bi-07 at 10^9^ cfu/mL as probiotics, 10 g of agave inulin as prebiotic, and the combination of both as synbiotic in antiretroviral-naïve, HIV-infected subjects.

There have been some studies in patients with HIV infection, using different probiotics in which mixed results have been obtained; this is perhaps secondary to the use of different concentrations and probiotic strains, which do not trigger the same immunostimulatory effect [15,31,32].

Certain serious adverse effects have been reported with the use of probiotics, particularly endocarditis, liver abscess, bacteremia, and septicemia or septic shock, especially in immunocompromised patients, or in patients with chronic disease, or in those with ventilatory support or with central venous catheters [33-35]. During the study, we received no report of serious adverse events, no clinically significant changes were noted in any safety parameter measured, and no patient developed sepsis, bacteremia, or acute inflammatory response. In addition, total bacterial load in feces decreased in probiotics and synbiotic groups and, the probiotics group showed an increase in the concentration of beneficial bacteria, as *Bifidobacterium* species, which have less potential for adhesion and translocation [12]. Further, we found that the synbiotic group had a tendency of reduction in beneficial and harmful bacterial loads and had a significant reduction in the concentration of bacterial translocation.

ARV treatment has increased survival in patients with HIV infection; however, survival is at least 10 years less than in general population. In addition, cardiovascular risk is twice as high in HIV-positive subjects [36]. In our study, the use of the synbiotic demonstrated a significant decrease in the concentration of cytokine IL-6, which has been utilized as a marker of cardiovascular risk, accelerated atherosclerosis, and mortality in HIV-infected patients [37]. However, it would therefore be important to assess, in our study, other cardiovascular risk-related parameters, such as d-dimer and high-sensitivity C-reactive protein [38].

ARV drugs can cause several adverse effects including dyslipidemia, insulin resistance, lactic acidosis, lipodystrophy, and diarrhea, among others [39,40]. Our study demonstrated a significant increase in CD4+ T lymphocyte levels in the synbiotic group, which could delay time of initiation of ARV treatment and decrease costs in countries with limited resources. Moreover, in our region, the agave pines of *Agave tequilana* Weber var*. azul* are widely employed to produce the national drink, tequila, and could also be a potential source of prebiotics and part of synbiotic with accessible prices.

On the other hand, we found no decrease in HIV-1 plasma viral load; therefore, the use of a synbiotic could not provide the benefit of maintaining an undetectable viral load as part of the primary prevention of HIV transmission.

## Competing interests

The authors have declared that no competing interests exist.

## Authors’ contributions

Conceived and designed the experiments: LAG, MFM, JFAV; Performed the experiments: LAG, LFJS; Analyzed the data: LAG, JFAV, LFJS; Contributed reagents/materials/analysis tools: MFM, AECL, LGVG, KEMS, LFEG; Wrote the paper: LAG, JFAV. All authors read and approved the final manuscript.
